# TACCO, a Database Connecting Transcriptome Alterations, Pathway Alterations and Clinical Outcomes in Cancers

**DOI:** 10.1038/s41598-019-40629-z

**Published:** 2019-03-07

**Authors:** Po-Hao Chou, Wei-Chao Liao, Kuo-Wang Tsai, Ku-Chung Chen, Jau-Song Yu, Ting-Wen Chen

**Affiliations:** 1 Molecular Medicine Research Center, Chang Gung University, Taoyuan, Taiwan; 2 Department of Otolaryngology-Head & Neck Surgery, Chang Gung Memorial Hospital, Linkou, Taiwan; 3 Center for General Education Chang Gung University, Taoyuan, Taiwan; 4 Department of Medical Education and Research, Kaohsiung Veterans General Hospital, Kaohsiung, Taiwan; 5 Department of Biochemistry and Molecular Cell Biology, School of Medicine, College of Medicine, Taipei Medical University, Taipei, Taiwan; 6 Department of Cell and Molecular Biology, Chang Gung University, Taoyuan, Taiwan; 7 Liver Research Center, Chang Gung Memorial Hospital, Linkou, Taiwan; 8 Institute of Bioinformatics and Systems Biology, National Chiao Tung University, Hsinchu, Taiwan

## Abstract

Because of innumerable cancer sequencing projects, abundant transcriptome expression profiles together with survival data are available from the same patients. Although some expression signatures for prognosis or pathologic staging have been identified from these data, systematically discovering such kind of expression signatures remains a challenge. To address this, we developed TACCO (Transcriptome Alterations in CanCer Omnibus), a database for identifying differentially expressed genes and altered pathways in cancer. TACCO also reveals miRNA cooperative regulations and supports construction of models for prognosis. The resulting signatures have great potential for patient stratification and treatment decision-making in future clinical applications. TACCO is freely available at http://tacco.life.nctu.edu.tw/.

## Introduction

Although considerable cancer sequencing data are already publicly available, systematically discovering meaningful correlations from these data is still challenging for cancer biologists lacking related computer skills. Large cancer sequencing projects, such as The Cancer Genome Atlas (TCGA), The International Cancer Genome Consortium (ICGC) and Therapeutically Applicable Research to Generate Effective Treatments (TARGET), have produced large amounts of sequencing data and made these data publicly available^1,2^. Although countless next-generation sequencing analysis tools and pipelines for processing high-throughput genomic and transcriptomic sequencing data have been developed, using these tools or pipelines still requires some basic command-line knowledge and sometimes even certain programming skills. Therefore, an easy-to-use interface that allows investigators to manage, integrate, and visualize cancer sequencing data across multiple cancer types without the need for computer skills would be a valuable tool for utilizing public cancer genomics data and advancing the cancer research field.

A number of databases, including FireBrowse, cBioPortal, OncoLnc, CancerMiner, GEPIA, miRCancerdb and MiRGator, are available for exploring transcriptome changes in cancers^3–8^. Using these databases, researchers can identify differentially expressed genes (DEGs), perform pathway analyses using these DEGs, explore correlations between expression levels of miRNAs and their target genes and analyze associations between the expression of individual genes and overall survival, among other functionalities. A five-miRNA (micro RNA) signature was recently proposed for stratification of patients with pancreatic adenocarcinoma into high-risk and low-risk groups with 5-year overall survival rates of 10.2% and 47.8%, respectively^9^. Similarly, other combined expression signatures have been proposed for lung adenocarcinoma, head and neck squamous cell carcinomas, glioblastomas, and breast cancers^10–13^. These signatures can potentially be used as clinical markers in personalized medicine; however, currently available databases only provide connections between the expression level of a single gene and survival data^3,6,7^. Therefore, a cancer transcriptome database that incorporates a feature that allows prognosis model construction would be extremely valuable.

In addition to survival signatures, another important, but often neglected, factor is miRNA-mRNA regulatory networks. Dysregulation of miRNA expression is significant in cancer formation and development^14^. miRNAs are 22-nucleotide long non-coding RNAs that target and regulate the expression of hundreds of target mRNAs; moreover, one gene may be targeted by multiple miRNAs. Thus, transcriptome alterations in cancer are a consequence of these multiple-to-multiple regulatory relationships among miRNAs and their target genes^15–17^. However, this type of combinatorial regulation of miRNAs has not been considered or investigated in previous cancer transcriptome databases. These miRNA cooperative modules can be taken into consideration by simply adding an analysis of how many miRNAs co-target the same genes. This additional information about such cooperative miRNAs can be helpful in selecting target genes for subsequent analysis or validation.

To fulfill all the analytical requirements for cancer transcriptomes, we propose the database, Transcriptome Alterations in CanCer Omnibus (TACCO). TACCO aims to provide an interactive interface that enables researchers to specify a group of significant differentially expressed miRNAs (DEmiRNAs) or DEGs, and subsequently perform pathway enrichment analysis and model construction for prognosis. TACCO will be useful for developing models for prognosis and thus should prove beneficial to the entire cancer research community.

## Results and Discussion

### Browse the expression levels of genes of interest in different cancer types

**Figure 1:**
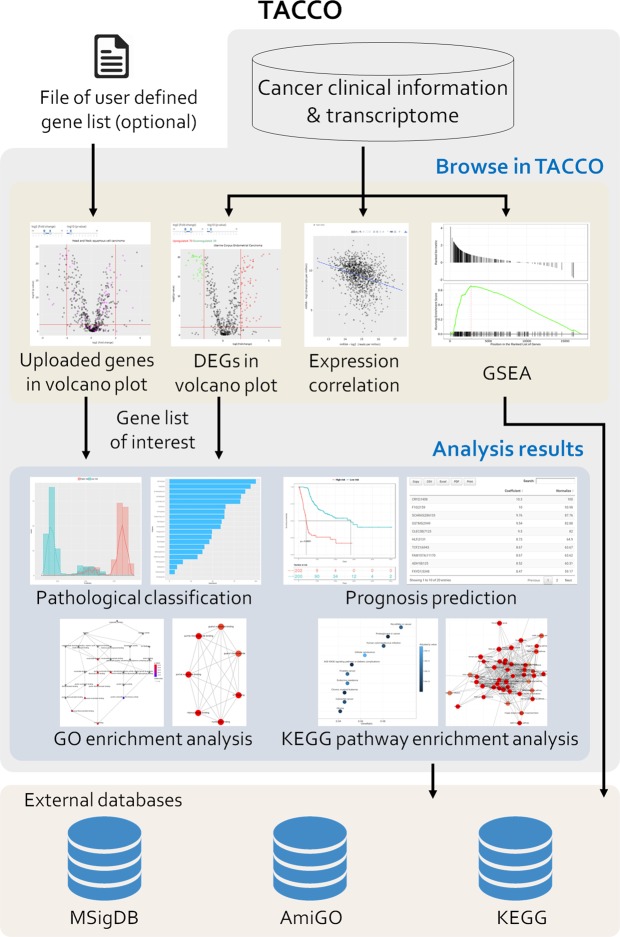
Overview of TACCO. TACCO was constructed using transcriptome data downloaded from several databases and provides GSEA results for gene sets from MSigDB, GO terms and KEGG pathways in 26 cancer types. In addition to GSEA, users can either identify DEGs/DEmiRNAs in TACCO or upload a gene set of interest. After a gene list is defined, users can construct a prediction model for pathological staging or clinical prognosis using the clinical or survival data in TACCO. For prognosis analysis, Kaplan-Meier survival plots with log-rank test p-values are provided for prediction results. Users can also perform a GO enrichment analysis, carry out KEGG pathway enrichment analysis^21^ and get the details of specific pathway by visiting external databases.

An overview of TACCO is shown in Fig. 1. TACCO provides gene and miRNA expression data for 26 and 22 cancer types, respectively. TACCO is the first cancer transcriptome database that includes miRNA-target correlations and provides the signature construction for prognosis and pathological staging. On the browse page, the user can either select or key in a gene symbol or miRNA ID of interest to explore expression fold changes, average expression levels in normal and tumor tissue, and p-values calculated from expression levels in tumor and adjacent normal tissues for different cancer types. TACCO also presents correlations between the expression levels of miRNA and target genes for cancer types for which both miRNA and gene expression data are available. While Pearson’s r and Spearman’s ρ are suitable for discovering linear correlation and rank correlation, respectably, both correlation analyses have been used in exploring miRNA-mediated regulation of target genes^5,8,18^. Therefore, TACCO calculates both Pearson’s r and Spearman’s ρ, and offers a distribution plot.

### Identify DEGs from a volcano plot

**Figure 2:**
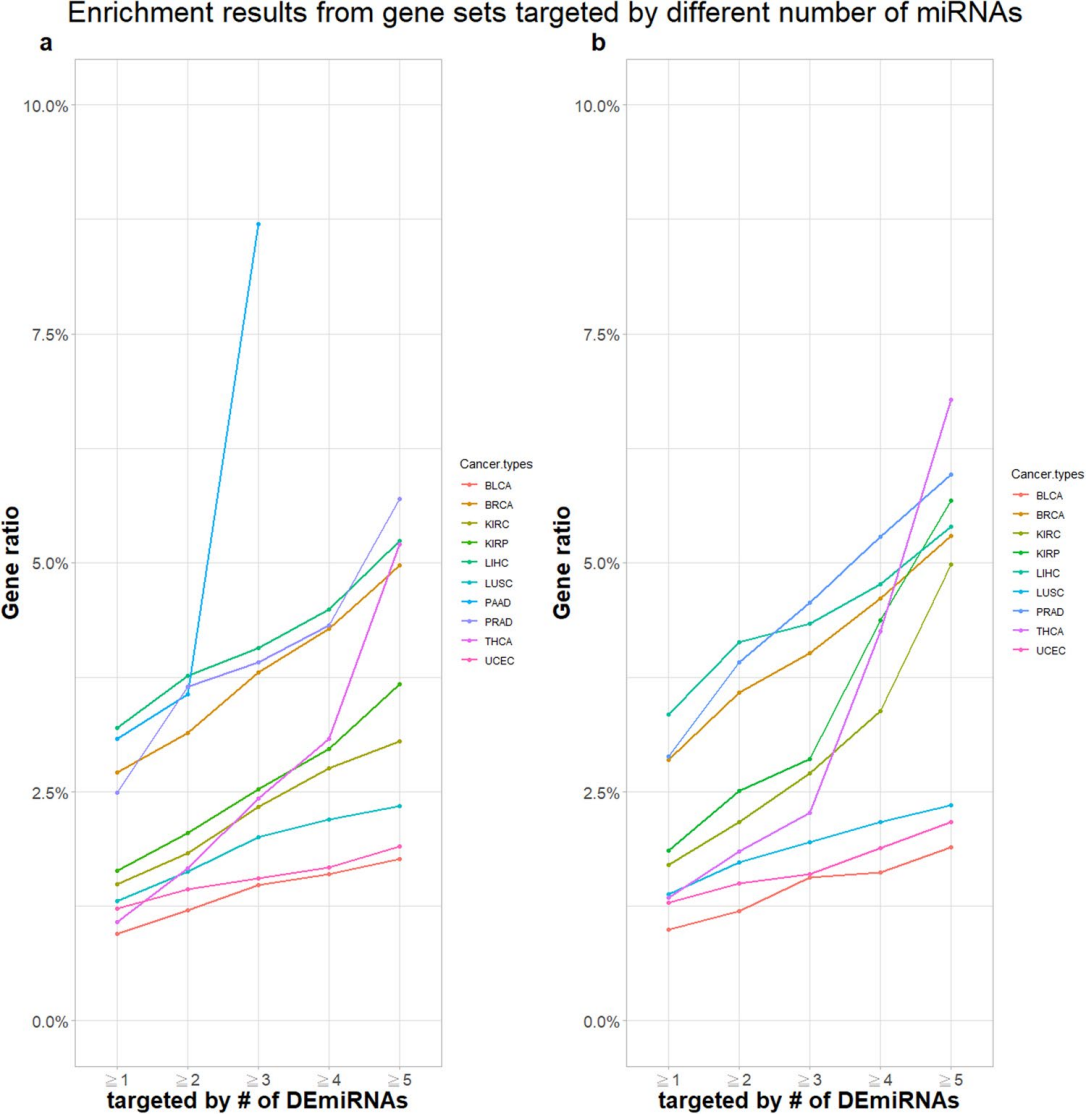
Genes targeted by a larger number of differentially expressed miRNAs (DEmiRNAs) are more frequently involved in cancer pathways. Both significantly upregulated and downregulated miRNAs were considered in (**a**), and only significantly upregulated miRNAs were considered in (**b**). For each cancer type, gene sets composed of genes targeted by different numbers of DEmiRNAs were used in KEGG pathway enrichment analyses. The percentage of genes involved in a given specific cancer type pathway were plotted. For example, in (A) a total of 92 genes are targeted by more than three DEmiRNAs in pancreatic cancer (PAAD; blue line), of which 8 were in KEGG hsa05212 (pancreatic cancer pathway); hence, the gene ratio is 8.7%. There is no additional data for PAAD because there are too few genes targeted by more than 4 or 5 DEmiRNAs for KEGG pathway enrichment analysis. Abbreviations: BLCA, bladder urothelial carcinoma; BRCA, breast invasive carcinoma; KIRC, kidney renal clear cell carcinoma; KIRP, kidney renal papillary cell carcinoma; LIHC, liver hepatocellular carcinoma; PAAD, pancreatic adenocarcinoma; LUSC, lung squamous cell carcinoma; PRAD, prostate adenocarcinoma; THCA, thyroid carcinoma; and UCEC, uterine corpus endometrial carcinoma.

TACCO illustrates transcriptome changes in the form of volcano plots together with slider bars for both p-values and fold changes. Hence, users can use customized criteria for identification of DEGs or look specifically for upregulated/downregulated gene lists. The volcano plots and number of DEGs refreshes on the fly upon user modification of the p-value or fold-change filter. After users apply their own criteria to identify a group of significant DEGs, these genes can be used in pathway enrichment analysis or model construction for survival prediction. TACCO also analyzes the number of DEmiRNAs that target the same gene, allowing users to investigate miRNA cooperative regulatory networks. To further investigate miRNA cooperative modules in cancers, we implemented KEGG pathway enrichment analysis in TACCO. We analyzed enriched pathways for genes co-targeted by at least 1, 2, 3, 4, or 5 DEmiRNAs for several cancer types which have related genes included in the KEGG database. We then calculated the percentage of genes among all targeted genes that have been reported in specific-cancer pathways. We found that the ratio of co-targeted genes that are found in the cancer pathways are positively correlated with the number of regulating DEmiRNA (Fig. 2). Although there are other cancer transcriptome databases, TACCO is the only one that provides information on miRNA co-target regulation. In our experience, the cooperative behavior of miRNAs—an important factor to consider in investigating alterations in the cancer transcriptome—may often be ignored.

### Specify enriched pathways in KEGG, MSigDB or GO categories

**Figure 3:**
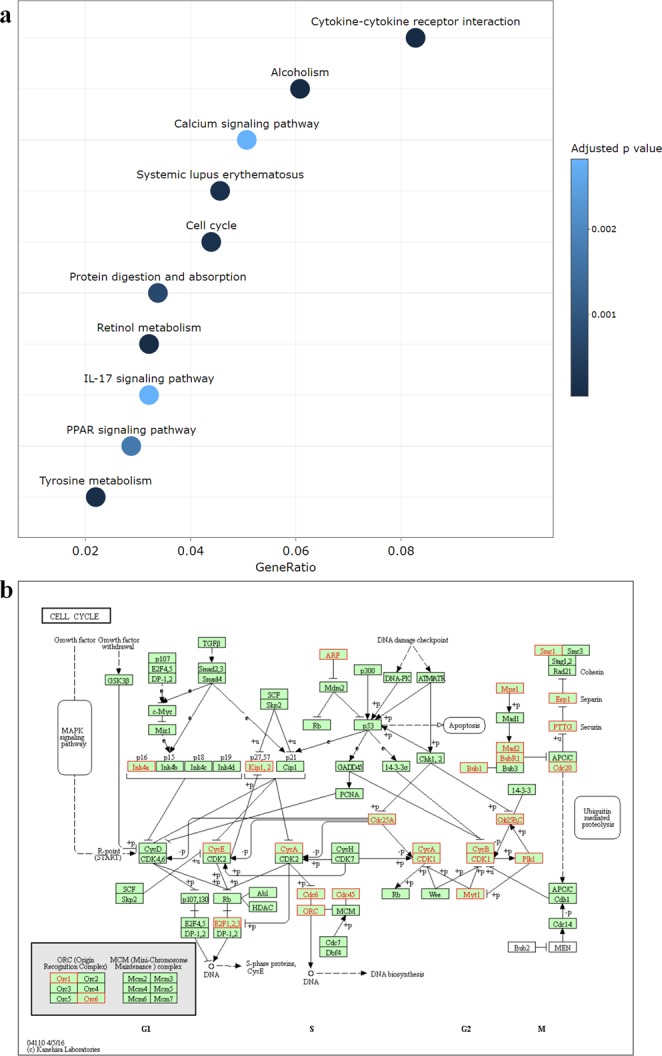
KEGG pathway enrichment results for differentially expressed genes (DEGs) in breast invasive carcinoma. (**a**) Enriched KEGG pathways are listed in a distribution plot in which the x-axis shows the ratio of genes included in DEGs for each pathway and the colors represent the adjusted enriched p-values. (**b**) TACCO provides links to KEGG pathway plots^21^. Here the enriched pathway cell cycle is shown where all the genes included in DEG list are written in red and highlighted in the red boxes.

**Figure 4:**
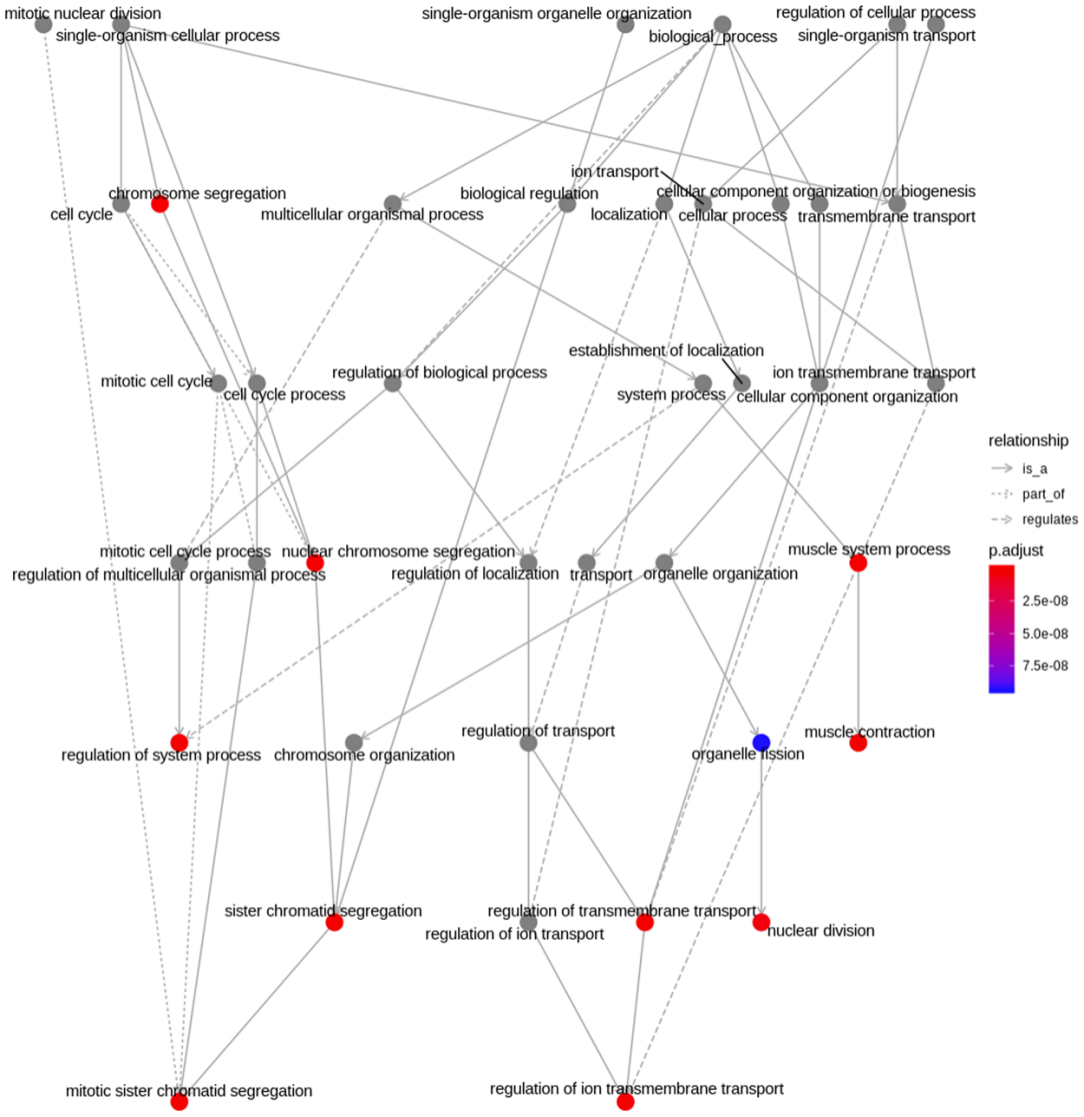
GO term enrichment result for differentially expressed genes (DEGs) in breast invasive carcinoma. The directed acyclic graph for overrepresented Biological process GO terms identified for DEGs are depicted. The circles represent GO terms, lines represent the relationship between GO terms and colors represent the adjusted enriched p-values.

For each cancer type, TACCO provides GSEA analysis for GO categories, gene sets from MSigDB and KEGG pathways^19–23^. Users can survey the enriched pathways together with the GSEA plot, normalized enrichment score adjusted p-value, and Q-value. Additionally, if DEGs were selected or users are interested in a specific gene list, TACCO also offers pathway enrichment analysis for subgroups of genes. TACCO utilizes a hypergeometric test to examine overrepresented pathways. For example, users can take the 803 up-regulated and 736 down-regulated genes (genes having absolute fold changes larger than 2 and p-values smaller than 0.01) in breast invasive carcinoma for the KEGG pathway and GO term enrichment analysis. KEGG pathway enrichment plots show the enrichment in many cancer-related pathways (Fig. 3a). TACCO also provides hyperlinks for all these pathways to the KEGG database and highlights the DEGs in the pathway (Fig. 3b). As for GO categories, TACCO provides a list of enriched GO terms and depicts a directed acyclic graph. The directed acyclic graph shows the parent-child relationships between GO terms and TACCO highlights the overrepresented GO terms (Fig. 4).

### Construct a model for prognosis or pathologic staging

**Figure 5:**
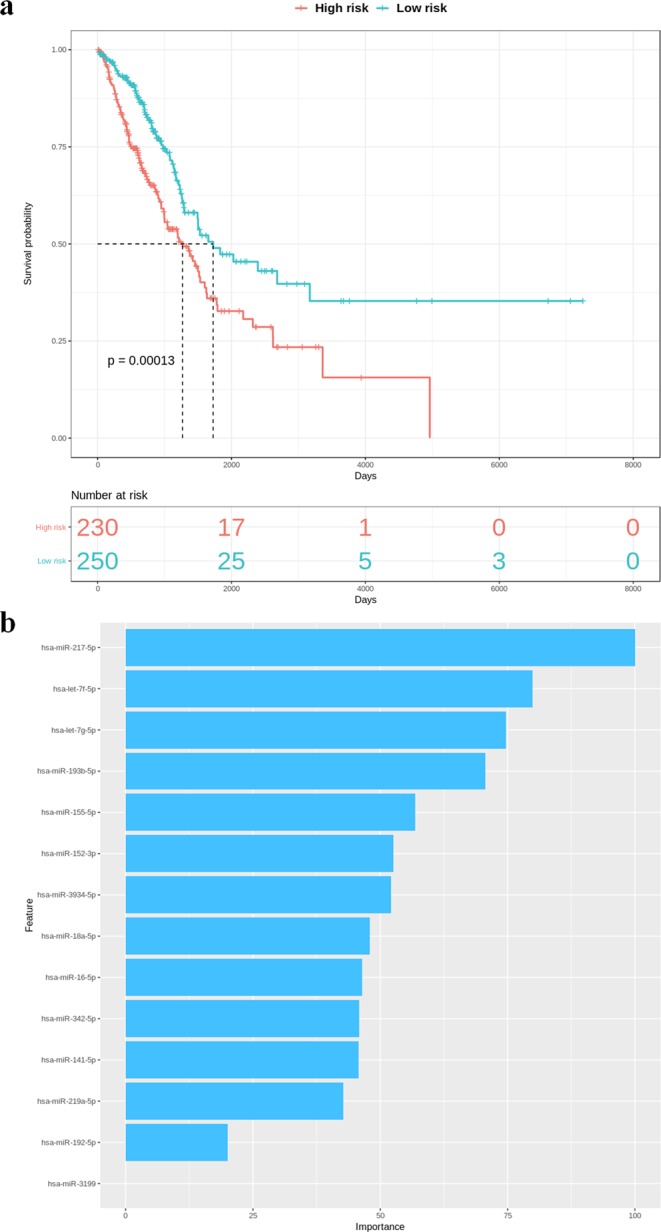
Model for prognosis constructed with TACCO. From a list of miRNAs TACCO constructs a prediction model for lung adenocarcinoma patients and uses this model to stratify samples into high and low risk groups. The patients from high and low risk were tested with log-rank test. TACCO provides tables and plots for the prediction results include (**a**) the Kaplan-Meier survival plot together with the number of patient surviving at each specific time point and (**b**) the importance for each predictor in the prediction model.

The significant DEGs identified in cancers are likely related to tumorigenesis; thus, their expression levels are potentially correlated with clinical outcome or cancer stage. Hence TACCO provides model construction for prognosis or pathologic staging. In addition to DEGs/DEmiRNAs, users can upload a specific gene list and select a cancer type for model construction. To identify a signature for prognosis, TACCO first evaluates the power of each gene or miRNA to distinguish patients with a good outcome from those with a bad outcome and then uses Lasso regression, Ridge regression, Classification and Regression Tree (CART), Random forest or Generalized Linear Models (GLM) to construct prediction models. TACCO also produces a Kaplan-Meier survival plot and log-rank p-value for the prediction results. For pathologic staging, TACCO evaluates the power of each gene or miRNA to distinguish patients from different cancer stage or TNM categories and then use aforementioned algorithms for prediction model construction. To illustrate the performance of TACCO, we tested two survival-associated miRNAs signatures provided from previous studies in pancreatic adenocarcinoma and lung adenocarcinoma^9,13^. We uploaded the reported list of miRNAs to TACCO and successfully constructed the prediction models which can distinguish low and high risk groups. As shown in Fig. 5, TACCO generates Kaplan-Meier survival plot for the predicted low and high risk groups from the 480 patients with lung adenocarcinoma as well as the importance of each predictor i.e. miRNA. Identification of a model or a signature that can predict overall or disease-free survival for various cancer types would be helpful, potentially guiding treatment decisions in the clinic.

In addition to GLM, TACCO utilizes Lasso regression and Ridge regression to identify transcriptome signatures for prognosis. Regression is one of the most commonly used machine-learning tasks, and the traditional and most popular of such methods are ordinary least squares regression and stepwise regression. However, both are known to be sensitive to random errors and are weak in terms of feature selection, prompting the development of Ridge regression and Lasso regression methods^24^. Lasso regression is a forward variable selection method that can choose one predictor out of a group of correlated variables. Lasso regression can also improve prediction accuracy in models with a limited number of predictors and provide better model interpretability. In addition, Lasso regression has recently been used to generate reliable models for survival prediction using transcriptome or protein expression data^25–27^. Thus, TACCO exploits Lasso regression for selection of transcriptome signatures and construction of prediction models for prognosis. For users who want to include all the uploaded or selected features in the prediction model, TACCO also provides Ridge regression. Meanwhile, decision tree based methods such as Random forest and CART are also included in TACCO because these two algorithms are also useful in signature construction for survival prediction^28,29^.

### An example–miR-17/92 miRNA cluster

**Figure 6:**
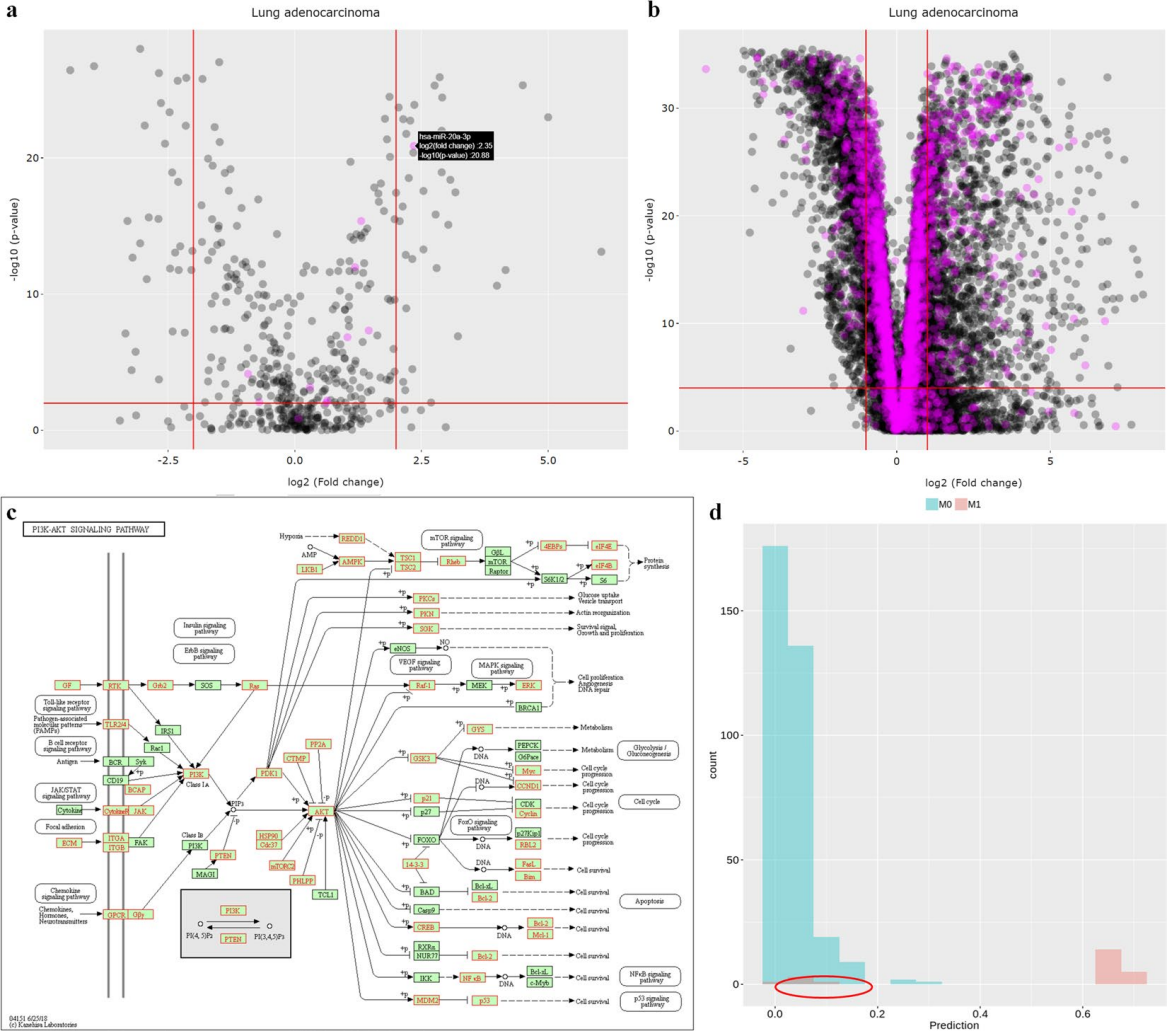
Explore the role of miR-17/92 miRNA cluster in lung adenocarcinoma with TACCO. (**a**) The volcano plot of miRNAs in lung adenocarcinoma. The miR-17/92 miRNA cluster includes 6 miRNAs, i.e. has-miR-17, hsa-miR-18a, hsa-miR-19a, hsa-miR-19b, hsa-miR-20a and hsa-miR-92a. The product of these 6 miRNAs are labeled in red. The most significantly upregulated miRNA is hsa-miR-20a-5p which has log2 fold change value equals to 2.35. (**b**) The volcano plot of genes in lung adenocarcinoma. The genes targeted by miR-17/92 miRNA cluster were labeled in purple. (**c**) PI3K-AKT signaling pathway is found enriched with genes targeted by the miRNAs located in miR-17/92 miRNA clusters. The targeted genes are in red box^21^. (**d**) The M stage (distant metastasis) classification results of the model constructed from miR-17/92 miRNA cluster and their target genes. The x-axis is the prediction values for classification. Patients with prediction value larger than 0.5 and smaller than 0.5 are predicted to be in M1 group and M0 group, respectively. The red and green color represent patients clinically diagnosed as from M1 and M0 groups, respectively. We found the prediction values for all M0 stage patients are smaller than 0.5 and hence correctly classified. As indicated by red circle, a few M1 stage patients have prediction values around 0.1 and wrongly classified as M0 group.

The oncogenic miR-17/92 miRNA cluster (has-miR-17, hsa-miR-18a, hsa-miR-19a, hsa-miR-19b, hsa-miR-20a and hsa-miR-92a) is known to be frequently overexpressed and play a prognostic role in lung cancer^30,31^. We used TACCO to investigate the role of miR-17/92 cluster in lung cancer. We first explored the negative regulation of miR-17/92 cluster on their target genes. For example, significantly negative correlation (p-value = 1.57*10^−13^, Spearman’s rank correlation) was found between the expression levels of has-miR-19b and PTEN and their spearman’s correlation coefficient is −0.321 in 510 lung adenocarcinoma samples. We found significant weak or moderate negative correlations between many miRNAs and their target genes which is expectable from heterogeneous tumor samples and also the complex transcriptome regulatory networks behind. We then uploaded a list of the all the miRNA products of miR-17/92 cluster and explored the expression changes of these miRNAs in lung adenocarcinoma. We found has-miR-20a-3p was 5.09 times up-regulated, this was consistent with previous studies^32,33^ (Fig. 6a). We also downloaded the targeted gene list of miR-17/92 cluster and then uploaded the gene list to TACCO to explore the expression changes of these targeted genes (Fig. 6b). We further carried out KEGG pathway enrichment with all the target genes for these miRNAs and found multiple cancer-related pathways enriched, including p53 signaling pathway, cellular senescence, proteoglycans in cancer and PI3K-Akt signaling pathway etc. We further explored the PI3K-Akt signaling pathway in KEGG database and found several important genes including AMPK, Ras, PI3K, Raf-1 and ERK are all targeted by the miR-17/92 miRNA cluster (Fig. 6c). We also tried to construct a model from these miRNAs to distinguish between patients with distant metastasis and without metastasis. Combining these miRNAs and their target genes, TACCO can provide a signature to differentiate patients with (M1) and without (M1) distant metastasis in lung adenocarcinoma. The signature composite of 121 genes include the top important TCP1, ARMT1, PIP4K2A which were already reported to correlated with metastasis in other cancer types^34–36^. Even though the signature misclassified few M1 as M0, most of the patients were correctly grouped (Fig. 6d).

### Comparison with other existing databases

Although other cancer transcriptome databases that come with interactive graphical user interfaces are available, TACCO is the only one that provides prediction model construction capability and information on cooperative miRNA modules (Table 1). As shown in this study, these cooperative regulatory interactions may be a non-negligible factor in studying transcriptome alterations. Four databases—MiRGator, GEPIA, cBioPortal and TACCO—provide identification of dysregulated genes or miRNAs, but only GEPIA and TACCO provide an interface that allows users to specify customized criteria for DEmiRNA or DEG identification. TACCO additionally provides volcano plots and the number of upregulated/downregulated DEGs, which refresh on the fly. Although FireBrowse, GEPIA and OncoLnc also offer survival analysis for single genes^3,7^, none of these databases provide model construction for survival prediction.

**Table 1:** Comparison of TACCO with other cancer transcriptome databases.

	TACCO	FireBrowse^#^	cBioPortal^#^	OncoLnc	GEPIA	CancerMiner	miRCancerdb	MiRGator
Pathway GSEA	✓	✓						
Identification of DEGs	✓		✓		✓			
Identification of DEmiRNA	✓		✓					✓
Correlation between the expression of miRNAs and target genes	✓	✓				✓	✓	✓
Pathway enrichment analysis	✓	✓	✓					✓
Survival analysis for single mRNAs/miRNAs		✓^*^		✓	✓^※^			
Analysis of multiple miRNAs that co-target an mRNA	✓							
Survival prediction model construction for multiple miRNAs and mRNAs	✓							

^#^Database for both genome and transcriptome.

^*^Only significant mRNA/miRNA are listed in the database.

^※^GEPIA provides survival analysis for mRNAs, but not miRNA.

## Conclusion

We propose a cancer transcriptome database, TACCO, that aims to link transcription alterations and transcriptome regulatory networks with alterations in downstream pathways and clinical outcomes in different cancer types. TACCO provides a user-friendly interface for assessing correlations between the expression of miRNAs and their target genes, identifying DEGs and altered pathways in cancers, and investigating miRNA co-target regulatory networks. Additionally, TACCO constructs models for prognosis from DEG lists or user-defined gene lists. Collectively, the analytical capabilities and model construction features present in TACCO make it feasible for researchers or clinicians to systematically investigate transcriptome regulatory network alterations and clinical outcomes in cancers. Accordingly, we believe that TACCO will shed light on important questions in the field of cancer research.

## Materials and Methods

### Identification of DEGs

Expression levels of miRNAs for 22 cancer types and mRNAs for 26 cancer types (Supplementary Table 1) were download from Broad GDAC Firehose version stddata__2016_01_28. All miRNA IDs were converted from MIMAT ID to miRBase nomenclature (hsa-miR-133a-3p, hsa-miR-557 etc.) based on miRBase 22 release^37^. Expression levels of mRNAs were obtained from RNAseqV2 data which were derived from RSEM^38^. For each cancer type, all genes with median expression levels greater than 0.01 transcript per million (TPM) across all samples were considered to be expressed. For each expressed gene, fold-change in expression levels between tumor and normal tissues, corresponding p-value, and Benjamini-Hochberg adjusted p-value were calculated using the EBSeq, Wilcoxon rank-sum test and multiple test correction in R^39,40^. Finally, volcano plots were generated using R package ggplot2. Based on validated interactions between miRNA and mRNA downloaded from miRTarBase 7.0^41^, TACCO also lists target genes for DEmiRNAs and calculates the number of DEmiRNAs that target these genes.

### Correlation between expression levels of mirna and target genes

For exploring regulatory relationships between miRNAs and their target genes, TACCO provides a tool that analyzes correlations between the expression levels of miRNAs and their target genes. When browsing TACCO, the user can select a specific gene or miRNA. TACCO calculates both parametric and non-parametric correlation coefficients (i.e., Pearson’s r and Spearman’s ρ) for expression levels of the interacting mRNA/target gene pair. In cases where a more normal expression distribution is needed, TACCO also offers correlation coefficients for log-transformed expression values. All correlation coefficients are listed in a table from which the user can select a gene or miRNA of interest to explore in detail. When the user selects a gene or miRNA from the table, a distribution plot for expression levels of the selected miRNA/target gene and a regression line with iteratively reweighted least squares is generated on the fly.

### Pathway enrichment analysis

TACCO provides Gene Set Enrichment Analysis (GSEA) for interpreting expression data for different cancer type^22^. TACCO also offers pathway enrichment analysis for either selected DEGs or an uploaded gene list. TACCO exploits the hypergeometric test, clusterProfiler, from the R package to identify enriched KEGG pathways or Gene Ontology (GO) terms^42^. TACCO generates a directed acyclic graph for enriched GO terms using R package, enrichplot. TACCO also displays a button to visit the KEGG website, where all the selected genes are highlighted within a red box.

### Identification of signatures for prognosis or pathological staging

All clinical and survival information for patients was downloaded with the R package, curatedTCGAData^43^. For each cancer type, all patients are divided into two groups based on their median survival/disease-free survival, in days. Differentially expressed mRNAs and miRNAs capable of distinguishing the two groups (Wilcoxon rank-sum test, p-value < 0.05) are selected as candidate features for signature identification^44^. Algorithms from the caret package^45^, including Lasso regression, Ridge regression, Random forest, Classification and Regression Tree (CART) and General Linear Model (GLM) were provided for prediction model construction. Prediction models constructed from 5-fold or 3-fold (for <150 patients) cross-validation is used to categorize patients into better (Low risk) or worse (High risk) surviving groups^45^. Finally, TACCO evaluates the prediction results using a Kaplan-Meier survival plot (KM plot) and log-rank test. Both KM plot and log-rank test results are generated to allow comparisons of survival data from predicted High-risk and Low-risk groups. TACCO also utilizes samples from early/late stage or different TNM stages to construct models for pathological staging with the same classification strategy.

### Website construction and availability

TACCO was built using Python, JavaScript, R and R Shiny on a Linux operating system and can be updated with new cancer sequencing projects based on current database schema. All tables and figures generated in TACCO can be downloaded by users. TACCO is available at http://tacco.life.nctu.edu.tw/ and can be explored with multiple web browsers, including Chrome, Internet Explorer, Firefox, and Safari.

## Supplementary information

Supplementary Table 1

## References

[1] Grossman RL (2016). Toward a Shared Vision for Cancer Genomic Data. N Engl J Med.

[2] Zhang J (2011). International Cancer Genome Consortium Data Portal–a one-stop shop for cancer genomics data. Database (Oxford).

[3] Tang Z (2017). GEPIA: a web server for cancer and normal gene expression profiling and interactive analyses. Nucleic Acids Res.

[4] Jacobsen A (2013). Analysis of microRNA-target interactions across diverse cancer types. Nat Struct Mol Biol.

[5] Cho S (2013). MiRGatorv3.0: a microRNA portal for deep sequencing, expression profiling and mRNA targeting. Nucleic Acids Res.

[6] Cerami E (2012). The cBio cancer genomics portal: an open platform for exploring multidimensional cancer genomics data. Cancer Discov.

[7] Anaya J (2016). OncoLnc: linking TCGA survival data to mRNAs, miRNAs, and lncRNAs. Peer J Computer Science.

[8] Ahmed M, Nguyen H, Lai T, Kim D (2018). R. miRCancerdb: a database for correlation analysis between microRNA and gene expression in cancer. BMC research notes.

[9] Shi XH (2018). A Five-microRNA Signature for Survival Prognosis in Pancreatic Adenocarcinoma based on TCGA Data. Scientific reports.

[10] Wong N (2016). Prognostic microRNA signatures derived from The Cancer Genome Atlas for head and neck squamous cell carcinomas. Cancer medicine.

[11] Volinia S, Croce CM (2013). Prognostic microRNA/mRNA signature from the integrated analysis of patients with invasive breast cancer. Proc Natl Acad Sci USA.

[12] Kim YW (2013). Identification of prognostic gene signatures of glioblastoma: a study based on TCGA data analysis. Neuro-oncology.

[13] Yerukala Sathipati S, Ho SY (2017). Identifying the miRNA signature associated with survival time in patients with lung adenocarcinoma using miRNA expression profiles. Scientific reports.

[14] Schickel R, Boyerinas B, Park SM, Peter ME (2008). MicroRNAs: key players in the immune system, differentiation, tumorigenesis and cell death. Oncogene.

[15] Shao T (2018). Survey of miRNA-miRNA cooperative regulation principles across cancer types. Brief Bioinform.

[16] Peter ME (2010). Targeting of mRNAs by multiple miRNAs: the next step. Oncogene.

[17] Chen WS (2013). Co-modulated behavior and effects of differentially expressed miRNA in colorectal cancer. BMC Genomics.

[18] Muniategui A, Pey J, Planes FJ, Rubio A (2013). Joint analysis of miRNA and mRNA expression data. Brief Bioinform.

[19] Liberzon A (2011). Molecular signatures database (MSigDB) 3.0. Bioinformatics.

[20] Liberzon A (2015). The Molecular Signatures Database (MSigDB) hallmark gene set collection. Cell Syst.

[21] Kanehisa M, Furumichi M, Tanabe M, Sato Y, Morishima K (2017). KEGG: new perspectives on genomes, pathways, diseases and drugs. Nucleic Acids Res.

[22] Subramanian A (2005). Gene set enrichment analysis: a knowledge-based approach for interpreting genome-wide expression profiles. Proc Natl Acad Sci USA.

[23] Ashburner M (2000). Gene ontology: tool for the unification of biology. The Gene Ontology Consortium. Nat Genet.

[24] Muthukrishnan, R. & Rohini, R. LASSO: A feature selection technique in predictive modeling for machine learning. *IEEE International Conference on Advances in Computer Applications* (2016).

[25] Zhang H (2016). Integrated Proteogenomic Characterization of Human High-Grade Serous Ovarian. Cancer. Cell.

[26] Ternes N, Rotolo F, Michiels S (2017). Robust estimation of the expected survival probabilities from high-dimensional Cox models with biomarker-by-treatment interactions in randomized clinical trials. BMC Med Res Methodol.

[27] Kaneko S, Hirakawa A, Hamada C (2015). Enhancing the Lasso Approach for Developing a Survival Prediction Model Based on Gene Expression Data. Comput Math Methods Med.

[28] Barlin JN (2013). Classification and regression tree (CART) analysis of endometrial carcinoma: Seeing the forest for the trees. Gynecol Oncol.

[29] Li J (2014). LncRNA profile study reveals a three-lncRNA signature associated with the survival of patients with oesophageal squamous cell carcinoma. Gut.

[30] Mogilyansky E, Rigoutsos I (2013). The miR-17/92 cluster: a comprehensive update on its genomics, genetics, functions and increasingly important and numerous roles in health and disease. Cell Death Differ.

[31] Liu F (2017). Prognostic role of miR-17-92 family in human cancers: evaluation of multiple prognostic outcomes. Oncotarget.

[32] Thompson TA (1996). Induction of apoptosis by organotin compounds *in vitro*: neuronal protection with antisense oligonucleotides directed against stannin. J Pharmacol Exp Ther.

[33] Osada H, Takahashi T (2011). let-7 and miR-17-92: small-sized major players in lung cancer development. Cancer Sci.

[34] Dainty, K. *Investigation into the Role of ARMT1 in Oestrogen Receptor Positive Breast Cancer*, University of Otago (2017).

[35] Coghlin C (2006). Characterization and over-expression of chaperonin t-complex proteins in colorectal cancer. J Pathol.

[36] Paula LM (2017). Analysis of molecular markers as predictive factors of lymph node involvement in breast carcinoma. Oncol Lett.

[37] Kozomara A, Griffiths-Jones S (2014). miRBase: annotating high confidence microRNAs using deep sequencing data. Nucleic Acids Res.

[38] Li B, Dewey CN (2011). RSEM: accurate transcript quantification from RNA-Seq data with or without a reference genome. BMC Bioinformatics.

[39] Benjamini Y, Hochberg Y (1995). Controlling the False Discovery Rate - a Practical and Powerful Approach to Multiple Testing. J Roy Stat Soc B Met.

[40] Leng N (2013). EBSeq: an empirical Bayes hierarchical model for inference in RNA-seq experiments. Bioinformatics.

[41] Chou CH (2018). miRTarBase update 2018: a resource for experimentally validated microRNA-target interactions. Nucleic Acids Res.

[42] Yu G, Wang LG, Han Y, He Q (2012). Y. clusterProfiler: an R package for comparing biological themes among gene clusters. OMICS.

[43] Ramos, M., Waldron, L., Schiffer, L., Obenchain, V. & Martin, M. curatedTCGAData: Curated Data From The Cancer Genome Atlas (TCGA) as MultiAssayExperiment Objects (2018).

[44] Friedman J, Hastie T, Tibshirani R (2010). Regularization Paths for Generalized Linear Models via Coordinate Descent. Journal of Statistical Software. Journal of Statistical Software.

[45] Kuhn M (2008). Building Predictive Models in R Using the caret Package. Journal of Statistical Software.

