# Supplementary material for: TACCO, a Database Connecting Transcriptome Alterations, Pathway Alterations and Clinical Outcomes in Cancers
Source: Sci Rep. 2019 Mar 7;9:3877. doi: 10.1038/s41598-019-40629-z (PMC6405743; doi:10.1038/s41598-019-40629-z)
Supplement: Supplementary file 1 — Supplementary Table 1 [file 41598_2019_40629_MOESM1_ESM.docx]

**TACCO, a Database Connecting Transcriptome Alterations, Pathway Alterations and Clinical Outcomes in Cancers**

Po-Hao Chou^1^, Wei-Chao Liao^1,2, 3^, Kuo-Wang Tsai^4^, Ku-Chung Chen^5^, Jau-Song Yu^1,6,7^, Ting-Wen Chen^8^**^*^**

*^3^Center for General Education Chang Gung University, Taoyuan, Taiwan.*

*^4^Department of Medical Education and Research, Kaohsiung Veterans General Hospital, Kaohsiung, Taiwan*

*^5^Department of Biochemistry and Molecular Cell Biology, School of Medicine, College of Medicine, Taipei Medical University, Taipei, Taiwan*

*^6^Department of Cell and Molecular Biology, Chang Gung University, Taoyuan, Taiwan.*

*^7^Liver Research Center, Chang Gung Memorial Hospital, Linkou, Taiwan.*

*^8^Institute of Bioinformatics and Systems Biology, National Chiao Tung University Hsinchu, Taiwan.*

^*^Corresponding author.

**Supplementary Table 1. Statistics on the number of samples in TACCO**

|  | mRNA | | miRNA | | | |  |
| --- | --- | --- | --- | --- | --- | --- | --- |
| Cancer types | Tumor | Normal | Tumor | | | Normal | |
| Bladder Urothelial Carcinoma | 408 | 19 | 414 | 19 | | |  |
| Breast invasive carcinoma | 1,093 | 112 | 1,091 | 104 | | |  |
| Cervical squamous cell carcinoma and endocervical adenocarcinoma | 304 | 3 | 307 | 3 | | |  |
| Cholangiocarcinoma | 36 | 9 | 36 | 9 | | |  |
| Esophageal carcinoma | 184 | 11 | 184 | 13 | | |  |
| Head and Neck squamous cell carcinoma | 520 | 44 | 523 | 44 | | |  |
| Kidney Chromophobe | 66 | 25 | 66 | 25 | | |  |
| Pan-kidney cohort (KICH+KIRC+KIRP) | 889 | 129 | 882 | 130 | | |  |
| Kidney renal clear cell carcinoma | 533 | 72 | 525 | 71 | | |  |
| Kidney renal papillary cell carcinoma | 290 | 32 | 291 | 34 | | |  |
| Liver hepatocellular carcinoma | 371 | 50 | 372 | 50 | | |  |
| Lung adenocarcinoma | 515 | 59 | 516 | 46 | | |  |
| Lung squamous cell carcinoma | 501 | 51 | 478 | 45 | | |  |
| Pancreatic adenocarcinoma | 178 | 4 | 178 | 4 | | |  |
| Pheochromocytoma and Paraganglioma | 179 | 3 | 179 | 3 | | |  |
| Prostate adenocarcinoma | 497 | 52 | 498 | 52 | | |  |
| Skin Cutaneous Melanoma | 103 | 1 | 97 | 2 | | |  |
| Stomach adenocarcinoma | 415 | 35 | 436 | 41 | | |  |
| Thyroid carcinoma | 501 | 59 | 502 | 59 | | |  |
| Thymoma | 120 | 2 | 124 | 2 | | |  |
| Uterine Corpus Endometrial Carcinoma | 545 | 35 | 542 | 33 | | |  |
| Stomach and Esophageal carcinoma | 599 | 46 | 620 | 54 | | |  |
| Colon adenocarcinoma | 457 | 41 | - | | - | | |
| Rectum adenocarcinoma | 166 | 10 | - | | - | | |
| Sarcoma | 259 | 2 | - | | - | | |
| Glioblastoma multiforme | 153 | 5 | - | | - | | |
